# Supplementary material for: Development and internal validation of the patient safety experience scale for inpatients
Source: PLoS One. 2025 Oct 7;20(10):e0332133. doi: 10.1371/journal.pone.0332133 (PMC12503294; doi:10.1371/journal.pone.0332133)
Supplement: S1 File — (DOCX) [file pone.0332133.s001.docx]

**Appendix 1.** **Patient Safety Experience Scale (PSES)**

| No. | | Items | strongly disagree | disagree | agree | strongly agree |
| --- | --- | --- | --- | --- | --- | --- |
| Patient identification | 1 | I was informed about the patient identification procedures carried out in the hospital. | ① | ② | ③ | ④ |
|  | 2 | I carefully checked whether the medical staff checked my name and registration number (or date of birth) during the medication/examination/surgery process. | ① | ② | ③ | ④ |
|  | 3 | I checked that my name was correct before wearing the patient identification bracelet. | ① | ② | ③ | ④ |
|  | 4 | I notified the medical staff when my patient identification bracelet was damaged and my name and registration number could not be verified. | ① | ② | ③ | ④ |
|  | 5 | I checked that the medication label attached to the injection matched my name. | ① | ② | ③ | ④ |
| Prevention of medication errors | 6 | I received sufficient explanation from my medical staff about the purpose of the medication and how to take it. | ① | ② | ③ | ④ |
|  | 7 | I immediately informed the medical staff when the medication (injection, etc.) was not administered properly. | ① | ② | ③ | ④ |
|  | 8 | I received sufficient explanation about the reason for the change when my medication was altered. | ① | ② | ③ | ④ |
|  | 9 | If I am not sure exactly how to take the medication (dosage, time, etc.), I ask the medical staff for explanation. | ① | ② | ③ | ④ |
|  | 10 | I immediately informed the medical staff if I experienced any abnormal symptoms (such as shortness of breath or dizziness) after taking the medication. | ① | ② | ③ | ④ |
| Fall prevention | 11 | I received regular training from medical staff on fall prevention. | ① | ② | ③ | ④ |
|  | 12 | I was instructed to notify the medical team promptly if I fell (falling out of bed, falling on the floor, etc.). | ① | ② | ③ | ④ |
|  | 13 | I received sufficient explanation about how to raise and lower the bed side rails. | ① | ② | ③ | ④ |
|  | 14 | When I had difficulty moving on my own, I asked the medical staff for help. | ① | ② | ③ | ④ |
|  | 15 | I wear shoes that have a firm, non-slip sole and fit my feet well. | ① | ② | ③ | ④ |
|  | 16 | I was instructed to sit in the wheelchair only after the wheels were securely locked | ① | ② | ③ | ④ |
|  | 17 | I was careful not to trip over the IV line when receiving an IV. | ① | ② | ③ | ④ |
| Infection prevention | 18 | I received sufficient explanation of infection prevention guidelines (hand washing, wearing a mask, etc.) while hospitalized. | ① | ② | ③ | ④ |
|  | 19 | I ensured that medical staff performed hand hygiene before administering treatment/medication. | ① | ② | ③ | ④ |
|  | 20 | I washed my hands with soap and water after blowing my nose or coughing. | ① | ② | ③ | ④ |
|  | 21 | I separated general waste and medical waste (alcohol swabs, gauze, etc) and disposed of them in the appropriate bins in the hospital room. | ① | ② | ③ | ④ |
|  | 22 | I changed my position periodically rather than staying in one position while I was awake. | ① | ② | ③ | ④ |
| Life safety compliance | 23 | I used medical equipment (beds, wheelchairs, crutches, etc) according to the instructions I was given. | ① | ② | ③ | ④ |
|  | 24 | I checked the location of the call bell in the hospital room and bathroom. | ① | ② | ③ | ④ |
|  | 25 | I checked the location of the evacuation facilities (guide lights, guide signs, evacuation routes, etc.). | ① | ② | ③ | ④ |
|  | 26 | I immediately informed the medical staff if any medical equipment (infusion pumps, monitors, etc) malfunctioned (e.g., alarms) or suddenly turned off. | ① | ② | ③ | ④ |
| Information sharing | 27 | I informed the medical staff if I had any discomfort related to treatment. | ① | ② | ③ | ④ |
|  | 28 | I received an explanation of the effects and side effects of the examination/treatment/procedure and signed a consent form. | ① | ② | ③ | ④ |
|  | 29 | I asked the medical staff any questions I had about the examination, treatment, or procedure. | ① | ② | ③ | ④ |
|  | 30 | I carefully read and checked the materials provided by the medical staff (hospital life guide, booklets, etc.). | ① | ② | ③ | ④ |
