# Supplementary material for: Development and internal validation of the patient safety experience scale for inpatients
Source: PLoS One. 2025 Oct 7;20(10):e0332133. doi: 10.1371/journal.pone.0332133 (PMC12503294; doi:10.1371/journal.pone.0332133)
Supplement: Attachment — Submitted filename: PONE-D-25-31484_reviewer.docx [file pone.0332133.s002.docx]

PLOS ONE

Development and Validation of the Patient Safety Experience Scale for Inpatients

--Manuscript Draft--

| Manuscript Number: | PONE-D-25-31484 |
| --- | --- |
| Article Type: | Research Article |
| Full Title: | Development and Validation of the Patient Safety Experience Scale for Inpatients |
| Short Title: | Development and Validation of the Patient Safety Experience Scale |
| Corresponding Author: | Sun-Hwa Shin Sahmyook University  Seoul , Seoul KOREA, REPUBLIC OF |
| Keywords: | Inpatients; patient safety; patient participation; reliability and validity |
| Abstract: | Purpose: This study aimed to develop and validate a scale to measure patient safety experiences among inpatients and address the growing need for standardized instruments to evaluate patient-centered safety practices. Methods: From an initial pool of 90 items derived from a literature review and qualitative interviews, 60 items were selected based on content validity assessment and expert review. A survey was conducted among 549 inpatients. Data were analyzed using item analysis, confirmatory factor analysis, Pearson’s correlation, Cronbach’s alpha, and intraclass correlation coefficients (ICC) using SPSS 26.0 and AMOS 21.0. Results: A two- dimensional, six-factor structure comprising 30 items was identified. These six factors were patient identification, prevention of medication errors, fall prevention, infection prevention, compliance with safety in daily life, and information sharing. The scale demonstrated excellent internal consistency (Cronbach’s α = .95) and strong test–retest reliability (ICC = .89). Additionally, it showed strong concurrent validity with the patient participation scale, with a correlation coefficient of .91. Conclusion: This validated 30-item scale is a reliable and robust instrument for assessing patient safety experiences among inpatients. It offers practical use in clinical settings by facilitating systematic evaluation and targeted improvements in patient safety, ultimately contributing to an enhanced quality of care and improved patient outcomes. |
| Order of Authors: | On-Jeon Baek |
|  | Sun-Hwa Shin |
| Additional Information: |  |
| Question | Response |
| Financial Disclosure  Enter a financial disclosure statement that describes the sources of funding for the work included in this submission. Review the [submission guidelines](http://journals.plos.org/plosone/s/submission-guidelines#loc-financial-disclosure-statement) for detailed requirements. View published research articles from [*PLOS ONE*](https://journals.plos.org/plosone/) for specific examples.  This statement is required for submission and will appear in the published article if the submission is accepted. Please make sure it is accurate. | The author(s) received no specific funding for this work. |

*Powered by Editorial Manager® and ProduXion Manager® from Aries Systems Corporation*

| Funded studies  Enter a statement with the following details:   - Initials of the authors who received each award - Grant numbers awarded to each author - The full name of each funder - URL of each funder website - Did the sponsors or funders play any role in the study design, data collection and analysis, decision to publish, or preparation of the manuscript? |  |
| --- | --- |
| Did you receive funding for this work? |  |
| Competing Interests  Use the instructions below to enter a competing interest statement for this submission. On behalf of all authors, disclose any [competing interests](http://journals.plos.org/plosone/s/competing-interests) that could be perceived to bias this work—acknowledging all financial support and any other relevant financial or non- financial competing interests.  This statement is required for submission and will appear in the published article if the submission is accepted. Please make sure it is accurate and that any funding sources listed in your Funding Information later in the submission form are also declared in your Financial Disclosure statement.  View published research articles from  [*PLOS ONE*](http://journals.plos.org/plosone/) for specific examples. | The authors have no conflicts of interest. |

*Powered by Editorial Manager® and ProduXion Manager® from Aries Systems Corporation*

| NO authors have competing interests  Enter: *The authors have declared that no competing interests exist.*  Authors with competing interests  Enter competing interest details beginning with this statement:  *I have read the journal's policy and the authors of this manuscript have the following competing interests: [insert competing interests here]* |  |
| --- | --- |
| * typeset |  |
| Ethics Statement  Enter an ethics statement for this submission. This statement is required if the study involved:   - Human participants - Human specimens or tissue - Vertebrate animals or cephalopods - Vertebrate embryos or tissues - Field research   Write "N/A" if the submission does not require an ethics statement.  General guidance is provided below. Consult the [submission guidelines](http://journals.plos.org/plosone/s/submission-guidelines#loc-guidelines-for-specific-study-types) for detailed instructions. Make sure that all information entered here is included in the Methods section of the manuscript. | The authors obtained human subject approval was obtained from the IRB (No. SYU 2023-12-008-003) before the beginning of the study. |

*Powered by Editorial Manager® and ProduXion Manager® from Aries Systems Corporation*

| Format for specific study types  Human Subject Research (involving human participants and/or tissue)   - Give the name of the institutional review board or ethics committee that approved the study - Include the approval number and/or a statement indicating approval of this research - Indicate the form of consent obtained (written/oral) or the reason that consent was not obtained (e.g. the data were analyzed anonymously)   Animal Research (involving vertebrate animals, embryos or tissues)   - Provide the name of the Institutional Animal Care and Use Committee (IACUC) or other relevant ethics board that reviewed the study protocol, and indicate whether they approved this research or granted a formal waiver of ethical approval - Include an approval number if one was obtained - If the study involved *non-human primates*, add *additional details* about animal welfare and steps taken to ameliorate suffering - If anesthesia, euthanasia, or any kind of animal sacrifice is part of the study, include briefly which substances and/or methods were applied   Field Research  Include the following details if this study involves the collection of plant, animal, or other materials from a natural setting:   - Field permit number - Name of the institution or relevant body that granted permission |  |
| --- | --- |
|  |  |
| Data Availability  Authors are required to make all data underlying the findings described fully available, without restriction, and from the time of publication. PLOS allows rare exceptions to address legal and ethical concerns. See the [PLOS Data Policy](http://journals.plos.org/plosone/s/data-availability) and [FAQ](http://journals.plos.org/plosone/s/data-availability#loc-faqs-for-data-policy) for detailed information. | Yes - all data are fully available without restriction |

*Powered by Editorial Manager® and ProduXion Manager® from Aries Systems Corporation*

| A Data Availability Statement describing where the data can be found is required at submission. Your answers to this question constitute the Data Availability Statement and will be published in the article, if accepted. |  |
| --- | --- |
| Important: Stating ‘data available on request from the author’ is not sufficient. If your data are only available upon request, select ‘No’ for the first question and explain your exceptional situation in the text box. |  |
| Do the authors confirm that all data underlying the findings described in their manuscript are fully available without restriction? |  |
| Describe where the data may be found in full sentences. If you are copying our sample text, replace any instances of XXX with the appropriate details.   - If the data are held or will be held in a public repository, include URLs, accession numbers or DOIs. If this information will only be available after acceptance, indicate this by ticking the box below. For example: *All XXX files are available from the XXX database (accession number(s) XXX, XXX.*). - If the data are all contained within the manuscript and/or Supporting Information files, enter the following: *All relevant data are within the manuscript and its Supporting Information files.* - If neither of these applies but you are able to provide details of access elsewhere, with or without limitations, please do so. For example:   *Data cannot be shared publicly because of [XXX]. Data are available from the XXX Institutional Data Access / Ethics Committee (contact via XXX) for researchers who meet the criteria for access to confidential data.*  *The data underlying the results presented in the study are available from (include the name of the third party* | All relevant data are within the manuscript and its Supporting Information files. |

*Powered by Editorial Manager® and ProduXion Manager® from Aries Systems Corporation*

| *and contact information or URL).*   - This text is appropriate if the data are owned by a third party and authors do not have permission to share the data.   * typeset |  |
| --- | --- |
| Additional data availability information: | Tick here if the URLs/accession numbers/DOIs will be available only after acceptance of the manuscript for publication so that we can ensure their inclusion before publication. |

*Powered by Editorial Manager® and ProduXion Manager® from Aries Systems Corporation*

<https://www2.cloud.editorialmanager.com/pone/download.aspx?id=40800618&guid=582aca12-c041-4048-bd38-48398ac6df62&scheme=1>Manuscript	[Click here to](https://www2.cloud.editorialmanager.com/pone/download.aspx?id=40800618&guid=582aca12-c041-4048-bd38-48398ac6df62&scheme=1)

[access/download;Manuscript;Manuscript_0612.docx](https://www2.cloud.editorialmanager.com/pone/download.aspx?id=40800618&guid=582aca12-c041-4048-bd38-48398ac6df62&scheme=1)

1 **Title page**
2 1) Title of the article:

# 3 Development and Validation of the Patient Safety Experience Scale for Inpatients

4

5 2) Author information: Names, affiliations, and ORCID numbers (required for all authors)

# 6 On-Jeon Baek

7 Part-time Lecturer, PhD, Eulji University College of Nursing, Gyeonggi-do, Korea
8 E-mail: [perfectoj@naver.com](mailto:perfectoj@naver.com)
9 ORCID: https://orcid.org/0000-0001-5057-406X

10

# 11 Sun-Hwa Shin

12 Associate Professor, PhD, Sahmyook University College of Nursing, Seoul, Korea
13 E-mail: [shinsh@syu.ac.kr](mailto:shinsh@syu.ac.kr)
14 ORCID: https://orcid.org/0000-0003-4052-9542

15

16 3) Corresponding author: Corresponding author's name, complete mailing address, email
17 address, and phone numbers
18 Name: **Sun-Hwa Shin**
19 Address: College of Nursing, Sahmyook University, 815 Hwarang-ro, Nowon-gu, Seoul,
20 South Korea, 01795.
21 E-mail: [shinsh@syu.ac.kr](mailto:shinsh@syu.ac.kr)
22 Tel: +82-2-3399-1595

23

24

# 1 Development and Validation of the Patient Safety Experience Scale for Inpatients

2

# 3 Abstract

4 **Purpose:** This study aimed to develop and validate a scale to measure patient safety
5 experiences among inpatients and address the growing need for standardized instruments to
6 evaluate patient-centered safety practices. **Methods:** From an initial pool of 90 items derived
7 from a literature review and qualitative interviews, 60 items were selected based on content
8 validity assessment and expert review. A survey was conducted among 549 inpatients. Data
9 were analyzed using item analysis, confirmatory factor analysis, Pearson’s correlation,
10 Cronbach’s alpha, and intraclass correlation coefficients (ICC) using SPSS 26.0 and AMOS
11 21.0. **Results:** A two-dimensional, six-factor structure comprising 30 items was identified.
12 These six factors were patient identification, prevention of medication errors, fall prevention,
13 infection prevention, compliance with safety in daily life, and information sharing. The scale
14 demonstrated excellent internal consistency (Cronbach’s α = .95) and strong test–retest
15 reliability (ICC = .89). Additionally, it showed strong concurrent validity with the patient
16 participation scale, with a correlation coefficient of .91. **Conclusion:** This validated 30-item
17 scale is a reliable and robust instrument for assessing patient safety experiences among
18 inpatients. It offers practical use in clinical settings by facilitating systematic evaluation and
19 targeted improvements in patient safety, ultimately contributing to an enhanced quality of
20 care and improved patient outcomes.

21

22	**Keywords:** Inpatients, patient safety, patient participation, reliability and validity

23

24

# 25 Introduction

26 Patient safety is a key factor in determining the quality of medical services by minimizing
27 unnecessary harm. The World Health Organization defines it as reducing risks associated
28 with healthcare to an acceptable minimum level [1–3]. The Korean Nurses’ Code of Ethics
29 states that patient safety should be given top priority in the nursing process [4]. The Korean
30 government is also strengthening patient safety policies and systematically evaluating patient
31 safety incidents and prevention activities through administrative data and reporting systems
32 [5]. However, to date, patient safety evaluations have mainly focused on medical
33 professionals, and there have been limitations in confirming safety incident experiences from
34 the patient’s perspective [1]. This highlights the need to evaluate and reflect on the safety
35 experiences of patients and develop patient-centered safety evaluation tools.
36 Recently, the Organization for Economic Cooperation and Development (OECD) has
37 made patient-centeredness a primary goal and has developed Patient-Reported Incidence
38 Measures (PRIMs), which enable patients to directly participate in reporting safety indicators
39 during the provision of healthcare services. Through these efforts, the OECD seeks to
40 systematically assess and improve patient safety experience [6]. “Patient safety experience”
41 is a concept that includes safety-related preventive activities, communication with healthcare
42 providers, and responses to incidents as directly perceived and recognized by patients [6].
43 To evaluate this, various indicators have been proposed, including the OECD’s PRIMs,
44 Agency for Healthcare Research and Quality (AHRQ), Patient Safety Indicators (PSIs) in
45 the United States, Joint Commission International (JCI), International Patient Safety Goals
46 (IPSG), and safety indicators from the Korea Institute for Healthcare Accreditation [6–9].
47 By measuring the safety experiences of inpatients based on these indicators, it is possible to
48 encourage patient participation in patient safety, prevent safety incidents, and use the results
49 as important data for evaluating and improving patient safety activities [10].
50 In response to the OECD’s recommendation that there was a lack of clear mechanisms to
51 ensure patient safety, the Health Insurance Review and Assessment Service in Korea has
52 developed a standard patient experience assessment questionnaire [11,12]. However,
53 although patient experience assessment is regarded as an important attempt to evaluate the
54 safety of healthcare services from the patient’s perspective, it has limitations in specifically
55 identifying the patient safety indicators presented by the OECD, AHRQ, and JCI [1]. To date,
56 patient safety measurement tools developed for inpatients include patient measures of safety
57 [13], patient-reported experiences and outcomes of safety in primary care [14], patient safety
58 perception [15], patient safety knowledge [16], patient participation [17], and performance
59 of patient safety activities [18]. Among these, the patient safety activity performance tool
60 developed by Kim and Park [18] includes some attributes of patient safety activities based
61 on the AHRQ patient safety indicators. However, other existing tools are limited to certain
62 aspects of patient safety. In addition, they do not reflect comprehensive attributes such as
63 experiences of participating in safety incident prevention activities, providing information,
64 and proactive safety activities. Therefore, to specifically assess the level of perception of
65 patient safety among inpatients, it is necessary to develop items based on patient safety
66 indicators and create a scale for safety experiences that are directly reported by inpatients.
67 Considering the limitations of existing tools, this study aims to develop a scale that reflects
68 the safety experiences of inpatients and is easy to use. To achieve this, we reviewed previous
69 literature and conducted in-depth, repeated explorations of the components of safety
70 experience among inpatients to derive the concept of patient safety experience. Through this
71 study, by systematically evaluating patient safety experiences, we sought to enhance the
72 understanding of patient safety experiences and ultimately contribute to promoting the
73 participation of inpatients in safety activities.
74 This study aimed to develop a patient safety experience scale that reflected patient safety
75 indicators for inpatients. The specific objectives were to (a) develop a patient safety
76 experience scale and (b) verify its reliability and validity.

77

# 78 Methods

79 **Research design**
80 This methodological study aimed to develop a scale to measure patient safety experiences
81 and assess its reliability and validity. This study follows the instrument development and
82 validation procedures proposed by DeVellis and Thorpe [19]. The methodological steps are
83 illustrated in Figure 1.

# 84 1. Scale development step

85 **(1) Constructing a concept definition**
86 To construct a conceptual framework for the patient safety experience, comprehensive
87 literature and in-depth interviews with hospitalized patients were conducted to identify its
88 key components. The literature review focused on publications between January 1, 2012, and
89 October 31, 2023, beginning in 2012 when the OECD initiated the development of patient-
90 reported patient safety indicators [20]. International literature was retrieved using the
91 keywords “patient safety,” “experience,” “scale,” “tool,” “instrument,” and “hospital”
92 through databases such as PubMed, CINAHL, Web of Science (WoS), Google Scholar, and
93 Embase. Korean literature was searched using the keywords “patient safety,” “experience,”
94 “tool,” “measurement,” “hospital,” and “medical institution” through RISS, NDSL, KMbase,
95 and DBpia. Based on the inclusion criteria, five international and eight Korean studies that
96 addressed the concept and attributes of inpatient patient safety experiences were selected.
97 Thirteen relevant studies were reviewed to derive the core attributes of the patient safety
98 experience.
99 In-depth interviews were conducted to gain a comprehensive understanding of inpatients’
100 experiences related to patient safety. Participants were purposively selected to ensure
101 diversity in characteristics such as age, sex, medical department, length of hospital stay, and
102 disease severity, including the inclusion of high-risk groups. The inclusion criteria were as
103 follows: age between 19 and 65 years, history of at least two hospitalizations, understanding
104 the study’s purpose, and prior consent to participate. Nine inpatients were interviewed and
105 recruited from five small and medium-sized hospitals (with fewer than 300 beds) located in
106 Seoul and the surrounding metropolitan area.
107 Nine participants (six women and three men) were interviewed, with an average of 2.6
108 prior hospitalizations. The interviews began with the question, “What do you think are the
109 safety factors experienced in medical institutions?” Additional questions included, “During
110 your hospitalization, what actions or environments provided by the medical staff or hospital
111 made you feel safe?”, “What activities or actions did you perform to ensure your safety
112 during hospitalization?”, and “Were there any factors that hindered your safety during
113 hospitalization?” Each interview lasted between 35 and 65 min. The collected data were
114 analyzed using the Krippendorff’s content analysis method [21].
115 Based on a comprehensive literature review and in-depth interviews, six key components
116 of patient safety experience were identified: patient identification, prevention of medication
117 errors, fall prevention, infection prevention, compliance with safety in daily life, and
118 information sharing. In this study, patient safety experience is defined as “safety-related
119 experiences that are directly perceived and recognized by inpatients from their own
120 perspective during the process of receiving medical care.”

# 121 (2) Initial items development and selection of a response format

122 Initial items were developed based on the conceptual attributes of the patient safety
123 experience. A total of 90 items were generated, consisting of 12 items for patient
124 identification, 14 for the prevention of medication errors, 16 for fall prevention, 16 for
125 infection prevention, 14 for compliance with safety in daily life, and 18 for information
126 sharing. A 4-point Likert scale suitable for measuring subjective perceptions was used to
127 assess opinions, beliefs, and attitudes [19]. To avoid central tendency bias, the scale excluded
128 neutral options and consisted of the following response categories: strongly disagree (1
129 point), disagree (2 points), agree (3 points), and strongly agree (4 points) [22]. Higher scores
130 indicated a greater level of patient safety experience, as perceived by inpatient respondents.

# 131 (3) Content validity

132 Content validity was assessed by convening a panel of experts to evaluate the
133 appropriateness of the initial items. Each item was rated on a 4-point Likert scale, and experts
134 provided qualitative feedback regarding the need to modify, supplement, add, or delete
135 specific items. The item-content validity index (I-CVI) was calculated by dividing the
136 number of experts who rated an item as “appropriate” or “very appropriate” by the total
137 number of experts. Items with an I-CVI of .78 or higher were retained. Additionally, the
138 scale-content validity index/average (S-CVI/Ave) was calculated by averaging the I-CVI
139 scores across all items with a threshold of .90 or higher, indicating acceptable content validity
140 at the scale level [22].
141 The first round of the content validity assessment was conducted between June 28 and
142 July 9, 2024. The expert panel consisted of nine professionals: two quality improvement (QI)
143 team managers from general hospitals, two nurse managers, three nursing professors with
144 expertise in tool development, one professor specializing in nursing administration, and one
145 professor of psychology. The I-CVI coefficients for the individual items ranged from 0.67 to
146 1.00, and the overall S-CVI/Ave was .90. Based on this analysis, 13 items with an I-CVI
147 below .78 and 18 items with redundant or overlapping content were removed. In total, 31
148 items were deleted, and 47 were revised according to expert feedback, resulting in 59 items
149 being retained for the next stage of validation.
150 The second content validity assessment was conducted between July 21 and July 25, 2024.
151 The expert panel consisted of seven members: six who participated in the first assessment
152 and one additional professor specializing in fundamental nursing. The I-CVI for the 59 items
153 ranged from .71 to 1.00, and the S-CVI/Ave was .95. Based on the results, two items, one
154 from the fall prevention domain and one from the information sharing domain, were removed
155 because the I-CVI values were below .78. In addition, three new items were added to the
156 infection prevention domain, based on expert recommendations. Following the second
157 assessment, a Korean linguist reviewed the items for grammar, vocabulary, spelling, and
158 sentence clarity, resulting in revisions to the wording of 11 items.
159 The third content validity assessment was conducted on August 5–14, 2024. The expert
160 panel comprised five members who participated in the second assessment and provided
161 feedback on item revisions. The I-CVI ranged from .80 to 1.00, and the S-CVI/Ave was .99.
162 Because all items met the validity criteria, no further deletions or modifications were made.
163 Consequently, a final total of 60 items were selected for the scale.

# 164 (4) Pilot survey

165 A pilot survey was conducted on August 17–24, 2024. To assess the comprehensibility and
166 difficulty of the 60 selected items, a survey was conducted with 20 adults who had been
167 hospitalized in the past year. Among the respondents, seven (35%) were men and 13 (65%)
168 were women, with an average age of 36.3 (±10.55) years. Sixteen (80%) participants
169 reported no underlying diseases. Regarding the number of hospitalizations, five (25%) were
170 hospitalized once, 11 (55%) were hospitalized two to three times, and four (20%) were
171 hospitalized four to five times.
172 The survey evaluated the font size, sentence comprehensibility, item difficulty, and time
173 required for completion. The participants were encouraged to comment on any items that
174 they found difficult to understand or required clarification. The average time to complete the
175 questionnaire was 10 min. The mean scores for comprehensibility, difficulty, and item length
176 appropriateness, which were rated on a 5-point Likert scale, were 4.35 (±.67), 4.25 (±.79),
177 and 4.15 (±.75), respectively. As no additional suggestions for revision were received, 60
178 items were finalized and used in the main survey.

| 179 |  |
| --- | --- |
| 180 | **2. Scale validation stage** |
| 181 | **(1) Participant and data collection for the main survey** |
| 182 | Participants in the main survey were conveniently sampled from five small- and medium- |
| 183 | sized hospitals (each with fewer than 300 beds) located in Seoul and the surrounding |
| 184 | metropolitan area. The inclusion criteria were as follows: patients aged 19–65 years, |
| 185 | hospitalized for at least three days, able to understand the purpose of the study, and who |
| 186 | provided informed consent. In total, 550 inpatients were surveyed. This number was |
| 187 | determined based on guidelines recommending 5–10 participants per item for exploratory |
| 188 | factor analysis (EFA) [19], and 200–400 participants for confirmatory factor analysis (CFA) |

189 using structural equation modeling. A 10% was added to account for potential dropouts [23].
190 Data for the main survey were collected between August 26 and September 18, 2024. The
191 researcher visited each participating hospital to explain the study’s purpose and procedures
192 to the head nurse or nursing manager and request institutional cooperation. The survey was
193 administered in both paper-based and online formats, depending on participants’ preferences.
194 The research assistants—nurses who were affiliated with each hospital—were trained in
195 advance regarding the study’s objectives and data collection procedures.
196 Participant recruitment was facilitated through posted notices in the hospitals. Individuals
197 who expressed an interest were included in the survey. In the paper-based version, the
198 research assistant explained the study’s background, objectives, participation, withdrawal
199 procedures, and potential risks and benefits. After obtaining written informed consent,
200 questionnaires were distributed and collected. The research assistant sent an online survey
201 link to the participants. Upon accessing the link, participants reviewed the “Research
202 Participant Information” and indicated informed consent by selecting “Understood” and “I
203 agree.”
204 The survey took approximately 15–20 min to complete. In total, 551 participants
205 responded: 149 via paper-based survey and 402 via online survey. After excluding two
206 incomplete responses, data from 549 participants were included in the final analysis. A
207 random sample of 300 was used for item analysis and EFA, whereas the remaining 249 were
208 used for CFA and concurrent validity testing.

# 209 (2) Evaluation of the scale: Construct validity

210 Construct validity was examined using SPSS (version 26.0) and AMOS (version 21.0
211 programs (IBM Corp., Armonk, NY, USA). For item analysis, the mean, standard deviation,
212 skewness, kurtosis, item-total correlation coefficient for each item, and the reliability
213 coefficient (Cronbach’s 𝘢) were computed when removing items. EFA was conducted by
214 confirming the suitability of the data using the Kaiser–Meyer–Olkin (KMO) test and
215 Bartlett’s test of sphericity. The maximum likelihood method and Promax rotation were used,
216 and items with a factor loading of less than .50 or a communality of less than .30 were deleted
217 [25].
218 CFA was performed to evaluate the model fit when the absolute fit index, χ^2^ value, and
219 degree of freedom ratio were 3 or less. The standardized root mean-square residual (SRMR)
220 and root mean square error of approximation (RMSEA) calculated using residuals were .08
221 or less, and the 90% confidence interval of the RMSEA was .10 or less. In addition, the
222 comparative fit index (CFI) and Turker-Lewis Index (TLI) values, which were incremental
223 fit indices, were .90 or higher as the criterion for appropriateness [23]. The standardized
224 factor coefficients (β) and modification indices (MI) were checked to modify the research
225 model. To verify the convergent validity of the items, the following criteria were applied:
226 standardized factor loading (β) of 0.50 or higher, average variance extracted (AVE) of 0.50
227 or higher, and construct reliability (CR) of 0.70 or higher [27]. Discriminant validity was
228 verified by confirming that the confidence interval of the correlation coefficient (Φ) (Φ ±
229 2.00 × SE) did not include 1.00. [26].

# 230 (3) Concurrent validity

231 Concurrent validity was assessed by examining its correlation with the patient
232 participation scale developed by Song and Kim [24], which addressed the components of
233 patient safety. The scale is a validated and reliable instrument consisting of 21 items across
234 four key subdomains of patient participation: sharing information and knowledge (eight
235 items), participation in the decision-making process (two items), engagement in proactive

| 236 | self-management activities (seven items), and establishing a mutually trusting relationship |
| --- | --- |
| 237 | (four items). Given that patient participation is a core component of the patient safety |
| 238 | experience, the conceptual relationship between the two instruments supports the rationale |
| 239 | for testing concurrent validity. Responses to the patient participation scale were rated on a 5- |
| 240 | point Likert scale ranging from 1 (strongly disagree) to 5 (strongly agree), with higher scores |
| 241 | indicating greater levels of patient participation during hospitalization. Cronbach’s α of the |
| 242 | scale was .92 in the original study [24] and .94 in the present study, demonstrating excellent |
| 243 | internal consistency. |
| 244 | **(4) Reliability test** |
| 245 | The homogeneity of reliability was verified by calculating Cronbach’s 𝘢. Stability was |
| 246 | assessed by calculating the intraclass correlation coefficients (ICC) to confirm test–retest |
| 247 | reliability. For the test–retest, 31 participants from the main survey who voluntarily agreed |
| 248 | to participate after being informed of the purpose and procedures of the survey completed |
| 249 | the same questionnaire two weeks later [19]. |
| 250 |  |
| 251 | **3. Ethical considerations** |
| 252 | Prior to data collection, approval was obtained from the Institutional Review Board (IRB |
| 253 | No: *** ****-12-008-003). All participants in the in-depth interviews, pilot survey, and main |
| 254 | survey were provided with an explanation document that included details of the purpose and |
| 255 | methods of the study, voluntary consent, decision to participate, benefits of participation, |
| 256 | information about the researchers, confidentiality, the possibility of withdrawal at any time, |
| 257 | and procedures for data storage and disposal. This information was thoroughly explained to |
| 258 | the participants. Participants in the online survey were provided a participant information |

259 sheet that included statements that the computerized survey data would be encrypted to
260 ensure security and accessibility only to the researcher, that anonymity would be guaranteed,
261 and that data would be permanently deleted after the completion of the study. Participants
262 who completed and submitted the survey received a token of appreciation, and those who
263 responded to the test–retest received two tokens of appreciation.

264

# 265 Results

266 **1. General characteristics of participants**
267 Among the 549 inpatients who participated in the main survey, 183 (33.3%) were men and
268 366 (66.7%) were women, with a mean age of 40.4 (±11.53) years. The largest age group
269 comprised those in their 30s (37.2%). In terms of marital status, 53.2% were married and
270 46.8% were unmarried. Most participants (77.1%) completed a university education, and
271 76.1% were employed. Most (63.6%) reported no underlying diseases, whereas 79.2% had
272 undergone procedures or surgeries. Regarding hospitalization history, 47.7% had been
273 hospitalized 2–3 times, and the most common department of admission was the internal
274 medicine department (57.6%). The most frequent length of hospital stay was 6–15 (51.4%)
275 days. Additionally, 85.4% had no experience of safety incidents, and 73.6% had received
276 patient safety education. No significant differences in the general characteristics were
277 observed between the participants assigned to the EFA (n = 300) and those assigned to the
278 CFA (n = 249) (Table 1).

279

# 280 2. Construct validity

281 Item means ranged from 3.14 to 3.50. Skewness (from −1.39 to −0.59) and kurtosis (from
282 −0.44 to 2.11) were within acceptable limits, indicating normality. The item-total correlation
283 coefficients ranged from .49 to .69. Reliability analysis showed a Cronbach’s α of .97 for all
284 60 items, which remained unchanged even when any item was removed; thus, all 60 items
285 were initially retained. As a result of the EFA, the KMO test of sampling adequacy was .95,
286 indicating excellent suitability for factor analysis. Bartlett’s test of sphericity was also
287 significant (χ² = 11,178, df = 1,770, p < .001), confirming the appropriateness of the
288 correlation matrix. A one-factor solution was also supported. Five items with communalities
289 below .30 were removed. The revised EFA, conducted with the remaining 55 items, showed
290 communalities ranging from .30 to .47; and all factor loadings were above .50
291 (range: .50–.71), indicating satisfactory item loadings on the extracted factor.
292 As a result of the CFA, the initial model showed a χ²/df value of 2.17, which was within
293 the acceptable range (<3). However, the model fit indices did not meet the recommended
294 thresholds (CFI = .77, TLI = .76, SRMR = .057, RMSEA = .069, 90% CI [.065, .072]). To
295 improve the model fit, standardized factor loadings (β) and MI were reviewed [23]. Items
296 with standardized loadings below .40 were removed. In addition, items with high MI values
297 between the measurement variables and error terms were eliminated if they overlapped
298 conceptually with other items or were prone to varied interpretations by respondents.
299 Iterative model refinement was performed using the maximum likelihood estimation method,
300 resulting in the removal of 26 items. The final model comprised 30 items across six factors:
301 Factor 1 (five items), Factor 2 (five items), Factor 3 (seven items), Factor 4 (five items),
302 Factor 5 (four items), and Factor 6 (four items). The revised model demonstrated acceptable
303 fit indices: χ²/df = 1.70, CFI = .91, TLI = .90, SRMR = .047, and RMSEA = .053 (90% CI
304 [.046, .060]).
305 Convergent validity analysis showed that standardized factor loadings ranged from .43
306 to .73, and the AVE values ranged from .48 to .57, with the AVE for Factor 1 falling below
307 the recommended threshold of .50. The CR values ranged from .80 to .90, with all factors
308 exceeding the acceptable criterion of .70. However, some confidence intervals for the inter-
309 factor correlation coefficients were 1.00, suggesting that discriminant validity was only
310 partially supported (Table 2).
311 The initial research model in this study assumed a one-dimensional six-factor structure
312 (Figure 2A), allowing for correlations among the latent variables. However, discriminant
313 validity was not fully supported because of the high inter-factor correlation coefficients.
314 When such high correlations are present and discriminant validity is compromised, either
315 simplifying the model into a single factor or considering a multidimensional structure is
316 recommended [27]. Therefore, two alternative models were tested using CFA: Alternative
317 Model I, a unidimensional single-factor structure (Figure 2B); and Alternative Model II, a
318 two-dimensional, six-factor structure (Figure 2C).
319 The model fit indices for Alternative Model I were χ²/df = 1.74, CFI = .90, TLI = .89,
320 SRMR = .048, and RMSEA = .055 (90% CI [.048, .061]). For Alternative Model II, the
321 indices were χ²/df = 1.68, CFI = .91, TLI = .90, SRMR = .047, and RMSEA = .052 (90% CI
322 [.046, .059]). Both models met the acceptable criteria for overall fit. A model comparison
323 using the Akaike Information Criterion (AIC) and Bayesian Information Criterion (BIC)
324 revealed lower AIC and BIC values for Alternative Model II, indicating that the two-
325 dimensional, six-factor model provided the best fit to the data (Table 2). Figure 2 shows the
326 standardized factor loadings for the initial and alternative models.

327

# 328 3. Concurrent validity

329 Results of the concurrent validity analysis showed that the correlation coefficient with the
330 patient participation scale (21 items) was .91 (p<.001), indicating a statistically significant
331 positive correlation. By subfactor, the correlations were as follows: patient identification .84
332 (p<.001), prevention of medication errors .80 (p<.001), fall prevention .81 (p<.001),
333 infection prevention .83 (p<.001), compliance with safety in daily life .83 (p<.001), and
334 information sharing .78 (p<.001), all of which showed statistically significant positive
335 correlations.

336

# 337 4. Reliability

338 The internal consistency of the scale, as measured by Cronbach’s α, was .95, indicating
339 excellent overall reliability. The subfactor reliability coefficients were as follows: patient
340 identification (.83), prevention of medication errors (.83), fall prevention (.87), infection
341 prevention (.83), compliance with safety in daily life (.83), and information sharing (.85), all
342 of which exceeded the acceptable threshold of .80.
343 Test–retest reliability was evaluated using ICCs based on data from 31 inpatients who
344 completed the scale twice at a two-week interval. The overall ICC was .89, confirming high
345 temporal stability. The sub-factors of ICCs were as follows: patient identification (.83),
346 prevention of medication errors (.63), fall prevention (.90), infection prevention (.83),
347 compliance with safety in daily life (.81), and information sharing (.72), demonstrating
348 acceptable to excellent stability across all domains.

349

# 350 5. Optimization of the scale

351 The patient safety experience scale developed in this study was finalized as a 30-item,
352 two-dimensional, six-factor structure based on comprehensive validity and reliability
353 evaluations. The scale uses a 4-point Likert response format ranging from 1 (“strongly
354 disagree”) to 4 (“strongly agree”), with higher scores indicating greater levels of patient-
355 perceived safety experience during hospitalization (Appendix 1).

356

# 357 Discussion

358 This study aimed to develop a scale to measure safety experiences that are directly
359 perceived and recognized by inpatients. Based on the six identified components, a 30-item
360 instrument with a two-dimensional, six-factor structure was developed. This structural model,
361 commonly used in disciplines such as social sciences, education, and psychology, enables
362 the evaluation of the overall patient safety experience through total scores or domain-specific
363 subfactor scores, providing flexibility in interpretation and application [28]. The scale is
364 significant because it reflects the core patient safety indicators proposed by internationally
365 recognized organizations, including the OECD, AHRQ, and JCI. Therefore, it offers a
366 systematic means of evaluating patient safety practices in clinical settings and has the
367 potential to contribute to the development of a patient safety culture.
368 In the six subfactors of patient safety experience, Factor 1 was “patient identification,”
369 which comprised five items and was associated with being informed about identification
370 procedures and cooperating with personal identification processes. These findings aligned
371 with those of Kim and Park [18], and similar domains were found in the nursing safety
372 activity scales for nurses, reinforcing the importance of accurate patient identification [29].
373 Patient identification is an essential first step in all medical procedures and plays a pivotal
374 role in ensuring safe healthcare delivery. In the study’s in-depth interviews, patients
375 acknowledged the potential risks of safety incidents when their personal information was not
376 properly verified and reported that they actively cooperated with repeated identification
377 checks conducted by healthcare staff. The items “I carefully checked whether the medical
378 staff verified my name and registration number (or date of birth) during medication,
379 examination, or surgery” and “When I received a patient identification bracelet, I checked if
380 the name was correct before wearing it” were retained as final items, allowing for the
381 evaluation of inpatients’ experiences with identification practices. Such active patient
382 involvement has been reported as a key factor in improving care quality and reducing patient
383 safety incidents [30]. The final scale incorporated the core standards for patient identification
384 proposed by the Korea Institute for Healthcare Accreditation [7], enhancing its practical
385 value for evaluating identification-related safety behaviors in clinical settings.
386 Factor 2 was “prevention of medication errors,” which comprised five items, such as
387 receiving explanations from medical staff about the purpose and effects of medications and
388 informing staff if an injection, and was omitted. This finding is consistent with that of
389 previous studies involving hospitalized patients, which emphasized patient safety behaviors,
390 such as being informed about drug efficacy, administration methods, side effects, and
391 precautions, as well as notifying healthcare providers in the event of abnormal infusion rates
392 [16,31]. Medication errors represent the second most common type of patient safety incidents
393 in healthcare settings, accounting for 31.9% of reported events, and their incidence continues
394 to increase annually, highlighting the need for effective prevention strategies [32,33].
395 Accordingly,	healthcare	professionals	must	provide	patients	with	comprehensive
396 explanations regarding the purpose, efficacy, and side effects of medications and rigorously
397 implement safety protocols during medication administration [34]. Moreover, a systematic
398 framework should be established to minimize medication errors by incorporating
399 institutional safeguards and patient involvement. Traditionally, prevention efforts have been
400 provider-centered; however, it is becoming increasingly important to actively engage
401 patients in these efforts. Educational programs aimed at improving patients’ communication
402 skills should be developed and implemented to empower them to ask questions about their
403 medications and promptly report abnormal reactions. This shift toward patient-centered
404 medication safety could enhance the overall safety culture in clinical practice.
405 Factor 3 was “fall prevention,” consisting of seven items, including receiving fall
406 prevention education and promptly reporting any fall incidents. Previous studies have
407 emphasized the importance of patient education and environmental interventions in
408 preventing falls among inpatients. Safety nursing activity scales also incorporate actions to
409 eliminate external risk factors, such as checking whether bed rails and wheelchair locks are
410 secure [35]. Furthermore, strategies such as fall risk assessment, patient education,
411 requesting staff assistance during movement, and environmental adjustments have been
412 identified as effective fall prevention measures [36,37], which align with the components
413 identified in this study. Falls can lead to severe outcomes, including mortality, and can
414 negatively affect patient outcomes by exacerbating health conditions and prolonging hospital
415 stay [37]. Studies have shown that patients at high risk of falling are influenced by multiple
416 factors, including medication side effects and physical and emotional health conditions,
417 requiring tailored interventions and patient education [36]. However, despite repeated
418 educational efforts, some patients fail to fully recognize the risk of falling. Therefore,
419 hospitals must implement ongoing and structured educational programs to raise patients’
420 awareness of the seriousness of falls and encourage active participation in fall prevention
421 practices.
422 Factor 4 was “infection prevention,” consisting of five items, including checking whether
423 medical staff performed hand hygiene and properly separated and disposed of medical waste.
424 Previous studies have identified the core elements of infection prevention, such as
425 compliance with hand hygiene guidelines, proper medical waste disposal, and respiratory
426 etiquette [8,19], which aligns with the results of this study. In-depth interviews further
427 revealed that patients emphasized the need for strict hand hygiene by medical staff during
428 invasive procedures, as well as detailed guidance regarding visitor restrictions. Such patient
429 education can enhance the awareness of infection prevention and patient safety, serving as
430 an important driver of desirable safety behaviors. Additionally, the prevention of pressure
431 ulcers, which can compromise the patients’ quality of life by extending hospital stay and
432 increasing healthcare costs, was considered. Medical institutions recommend integrated
433 strategies for pressure ulcer prevention, including nutritional assessments and scheduled
434 position changes [38]. Accordingly, the item “I changed my body position periodically rather
435 than staying in one position while awake” was included to reflect this aspect. This suggests
436 that pressure ulcer prevention is not only a key element of infection control but also
437 contributes to patient comfort and overall safety.
438 Factor 5 was “life safety compliance,” consisting of four items, including checking the
439 location of the call bell and notifying staff in the event of medical equipment malfunction.
440 This domain is consistent with that of previous studies that emphasize the importance of
441 refraining from arbitrarily handling medical devices, knowing how to call for help in
442 emergencies, and adhering to fire safety protocols [18,29,35]. Such daily safety compliance
443 plays a crucial role in minimizing environmental risk factors and ensuring the safety of not
444 only patients but also caregivers and visitors. Medical institutions typically provide regular
445 safety education focused on fire prevention, prohibit the operation of medical equipment by
446 nonclinical personnel, and encourage prompt reporting of equipment malfunctions, thereby
447 underscoring the importance of patient safety [39]. In this study, items were developed to
448 reflect safety behaviors that required the active participation of patients, such as identifying
449 the location, using of call bells, and recognizing emergency evacuation signage. These
450 findings suggest that life safety compliance not only helps prevent safety incidents but also
451 reinforces the patient’s proactive role in maintaining a safe healthcare environment.
452 Factor 6 was “information sharing,” which comprised four items, including asking
453 questions about areas of curiosity and reading educational materials provided by medical
454 staff. In this study, information sharing was conceptualized as extending beyond the simple
455 transmission of information to include patient engagement in treatment decisions and the
456 cultivation of trust-based relationships with healthcare providers. Previous studies have
457 emphasized that effective communication is a fundamental component of patient safety,
458 contributing to the reduction of medical errors and enhancement of care quality [2,17,40].
459 When a trusting relationship is established, patients are more likely to actively participate in
460 safety-related behaviors [24] and engage in shared decision-making, taking greater
461 ownership of their health management [41]. However, findings from in-depth interviews
462 indicated that patients often struggled to understand the need of their treatment owing to the
463 use of complex medical terminology and brief, task-oriented explanations by healthcare
464 professionals. This communication barrier limits patients’ ability to ask questions or seek
465 clarification, an observation supported by earlier studies reporting that time constraints
466 among medical staff can impede patient-centered communication [24,42]. To enhance
467 patient engagement, healthcare professionals should improve communication strategies; and
468 systemic efforts, such as reducing administrative burdens, are required to support effective
469 interactions.
470 The average score on the patient safety experience scale developed in this study was 3.32
471 of 4, with most subfactors exceeding 3.30. This indicates that hospitalized patients generally
472 perceived and experienced a relatively high level of safety during their hospital stay.
473 However, the scale does not include safety domains associated with special clinical situations

| 474 | such as blood transfusions or the specific needs of high-risk patients. Therefore, caution |
| --- | --- |
| 475 | should be exercised when applying this instrument to all inpatients. Nevertheless, the scale |
| 476 | is meaningful because it allows healthcare institutions to identify and reevaluate vulnerable |
| 477 | areas in patient safety practices from a patient’s perspective. Furthermore, it encourages |
| 478 | patient engagement in safety-related behaviors, thereby contributing to the development of |
| 479 | patient-centered safety strategies. This instrument may provide foundational data for |
| 480 | improving institutional safety protocols and ultimately supporting the establishment of a |
| 481 | culture of safety that centers on patients. |
| 482 |  |
| 483 | **Conclusion** |
| 484 | This study developed and validated a patient safety experience scale that reflected core |
| 485 | indicators from organizations such as the AHRQ, JCI, OECD, and the Korea Institute for |
| 486 | Healthcare Accreditation. The final tool, consisting of 30 items across six sub-factors in a |
| 487 | two-dimensional structure, demonstrated strong reliability and construct validity. Designed |
| 488 | for ease of use in clinical settings, the scale enables quantitative evaluation of inpatients’ |
| 489 | safety-related experiences. Based on the findings of this study, future research should aim to |
| 490 | validate the patient safety experience scale across a broader range of clinical settings, |
| 491 | including general and tertiary hospitals in diverse regions to assess its generalizability and |
| 492 | cross-validity. Considering the insufficient discriminant validity owing to high inter-factor |
| 493 | correlations in the CFA, further conceptual clarification of patient safety experiences through |
| 494 | additional analysis is warranted. Intervention studies using the developed scale are |
| 495 | recommended to evaluate and enhance patient safety-related behaviors and experiences in |
| 496 | clinical practice. |

497

# 498 CONFLICTS OF INTEREST

499	The authors declared that no conflict of interest.

500

# 501 ACKNOWLEDGEMENTS

502	None.

503

# 504 DATA SHARING STATEMENT

505	Please contact the corresponding author for data availability.

506

507

# 508 REFERENCES

509 1. Im J, Kim KY, Dong J, Lee PH, Ock M. Applicability of OECD patient-reported incidence
510 measures that reflect the perspective of healthcare professionals and patients: mixed
511 methods	research.	Korean	Soc	Qual	Health	Care.	2023;29(1):2-14.
512 https://dx.doi.org/10.14371/QIH.2023.29.1.2
513 2. Institute of Medicine (IOM). Crossing the quality chasm: a new health system for the 21st
514 century. Washington, D.C. National Academies Press; 2001.
515 3. World Health Organization. Patient safety. Geneva: World Health Organization; 2023
516 https:/[/www.who.int/news-room/fact-sheets/detail/patient-safety](http://www.who.int/news-room/fact-sheets/detail/patient-safety)
517 4. Korea Nursing Association. Korean Nurses code of ethics [Internet]. Korea Nursing
518 Association;	c2023	[cited	2024	November	30].	Available	from:
519 [http://www.koreanurse.or.kr/about_KNA/ethics.php?mn=3#a18](http://www.koreanurse.or.kr/about_KNA/ethics.php?mn=3&a18)
520 5. Kwak MJ, Park SH, Kim CG, Park T, Lee SI, Lee SG, et al. Strategies and experts in other
521 countries for patient safety and quality improvement. Korean Soc Qual Health Care.
522 2020;26(2):104-112. <http://dx.doi.org/10.14371/QIH.2020.26.2.104>
523 6. Organization for Economic Co-operation and Development (OECD). Patient-reported safety
524 indicators: question set and data collection guidance. OECD; c2019 [cited 2024
525 November	1].	Available	from:	https:/[/www.oecd.org/health/health-care-quality-](http://www.oecd.org/health/health-care-quality-)
526 outcomes-indicators.htm
527 7. Korea Institute for Healthcare Accreditation. Announcement of the 4th cycle acute care
528 hospital accreditation criteria and standard guidelines [Internet]. Korea Institute for
529 Healthcare	Accreditation;	c2021.	Available	from:
530 https:/[/www.koiha.or.kr/web/kr/library/establish_view.do](http://www.koiha.or.kr/web/kr/library/establish_view.do)
531 8. Agency for Healthcare Research and Quality (AHRQ). 20 Tips to help prevent medical errors.
532 c2011. Available	from:	https:/[/www.ahrq.gov/sites/default/files/wysiwyg/patients-](http://www.ahrq.gov/sites/default/files/wysiwyg/patients-)
533 consumers/careplanning/errors/20tips/20tips.pdf
534 9. Joint Commission International (JCI). International Patient Safety Goals. JCI; c2024 [cited
535 November	1].	Available	form:
536 https:/[/www.jointcommissioninternational.org/standards/international-patient-safety-](http://www.jointcommissioninternational.org/standards/international-patient-safety-)
537 goals/
538 10. Lee HM, Chun JH. The significance of Joint Commission International accreditation. J

539	Korean Med Assoc. 2012;55(1):17-22. <http://dx.doi.org/10.5124/jkma.2012.55.1.17>

540 11. Organization for Economic Co-operation and Development (OECD). OECD reviews of
541 health care quality: Korea – Raising Standards, OECD Publishing. c2012. Available
542 from: <http://dx.doi.org/10.1787/9789264173446-en>
543 12. Do YK, Kim JE, Lee JY, Lee HY, Cho MW, Kim EN et al. Development of a patient-
544 centered evaluation model. Health Insurance Review & Assessment Service; 2015.
545 Report No.: G000E70-2015-87.
546 13. Giles SJ, Lawton RJ, Din I, McEachan RR. Developing a patient measure of safety (PMOS).

547	BMJ Qual & Saf. 2013;22(7):554-562. https://doi.org/10.1136/bmjqs-2012-000843

548 14. Ricci-Cabello I, Avery AJ, Reeves D, Kadam UT, Valderas JM. Measuring patient safety in
549 primary care: the development and validation of the “Patient Reported Experiences and
550 Outcomes of Safety in Primary Care”(PREOS-PC). Ann Fam Med. 2016;14(3):253-
551 261.
552 15. Kim KJ, Lee EH, Shin SH. Development and validation of the patient safety perception
553 scale	for	hospitalized	patients.	Korean	J Adult	Nurs.	2018;30(4):404-416.
554 https://doi.org/10.7475/kjan.2018.30.4.404
555 16. Lee JW, Park MH. Perception, knowledge and attitudes of hospitalized patients toward
556 patient	safety.	J	Korean	Soc	Nurs	Ethics.	2022;2(2):11-20.
557 https://doi.org/10.51462/jksne.2022.2.2.11
558 17. Kang SJ, Park JY. Patient safety perception and patient participation among hemato-
559 oncology	patients.	Asian	Oncol	Nurs.	2019;19(4):224-232.
560 https://doi.org/10.5388/aon.2019.19.4.224
561 18. Kim AN, Park JS. Awareness of patient safety and performance of patient safety activities
562 among hospitalized patients. J Korea Acad-Indu Cooper Soc. 2021;22(5):229-240.
563 https://doi.org/10.5762/KAIS.2021.22.5.229
564 19. DeVellis RF, Thorpe CT. Scale development: Theory and applications 5th ed. Sage
565 publications; 2022. 320p.
566 20. Ministry of Health & Wellfare. Production and development of OECD health care quality
567 indicators as of 2012. Health Insurance Review & Assessment Service; 2014. Report
568 No.: 11-1352000-000759-10.
569 21. Krippendorff K. Content analysis: An introduction to its methodology 4th ed. Sage
570 publications; 2018. 472p.
571 22. Lynn MR. Determination and quantification of content validity. Nurs Res. 1986;35(6):382-

572	386.

573	23. Hair JF, Black WC, Babin BJ, Anderson RE. Multivariate data analysis: A global

574	perspective 7th ed. Pearson Education Upper; 2016. 800p.

575	24. Song M, Kim M. Development and validation of a patient participation scale. J Adv Nurs.

576	2023;79(6):2393-2403. https://doi.org/10.1111/jan.15593

577	25. Sivo SA, Fan X, Witta EL, Willse JT. The search for “optimal" cutoff properties: Fit index

578	criteria	in	structural	equation	modeling.	J	Exper	Edu.	2006:74(3):267-288.

579	https://doi.org/10.3200/JEXE.74.3.267-288

580	26. Woo JP. Understanding of structural equation modeling and its concepts. Hannarae

581	Publisher; 2016. p.1-568.

582	27. Brown TA. Confirmatory Factor Analysis for Applied Research. New York: Guilford Press;

583	2015. 462p.

584 28. Kumlander S, Lahtinen O, Turunen T, Salmivalli C. Two is more valid than one, but is six
585 even better? The factor structure of the Self-Compassion Scale (SCS). PLoS One.
586 2018;13(12):e0207706. https://doi.org/10.1371/journal.pone.0207706
587 29. Han MY, Jung MS. Effect of hospital nurses' perceptions of organizational health and
588 patient safety culture on patient safety nursing activities. J Korean Acad Nurs Adm.
589 2017;23(2):127-138. <http://dx.doi.org/10.11111/jkana.2017.23.2.127>
590 30. Yu C, Xian Y, Jing T, Bai M, Li X, Li J, et al. More patient-centered care, better healthcare:
591 the association between patient-centered care and healthcare outcomes in inpatients.
592 Front Public Health. 2023;11:1-9. https://doi.org/10.3389/fpubh.2023.1148277
593 31. KOrea Patient Safety reporting & learning system (KOPS). ‘Three promises for safe
594 medication’ with patients and caregivers [Internet]. KOPS; c2022 [cited 2024
595 November	1].
596 https:/[/www.kops.or.kr/portal/ifm/infoProvdStdrDetail.do?infoProvdNo=31](http://www.kops.or.kr/portal/ifm/infoProvdStdrDetail.do?infoProvdNo=31)
597 32. Ministry of Health and Welfare, Korea Institute for Healthcare Accreditation. (2022). 2021
598 Patient Safety Statistics Yearbook [Internet]. KOPS; c2022 [cited 2024 November 1].
599 https:/[/www.kops.or.kr/portal/board/statAnlrpt/boardDetail.do](http://www.kops.or.kr/portal/board/statAnlrpt/boardDetail.do)
600 33. Yoon SH. Factors related to medication errors in hospitals. Korean Assoc Learn-Cent Curric

601	Instr. 2022;22(17):787-796. https://doi.org/10.22251/jlcci.2022.22.17.787

602	34. Kim J, Song Y, Suh SR. The predictive factors of medication errors in clinical nurse. J

603	Health Info Stat. 2021;46(1):19-27. https://doi.org/10.21032/jhis.2021.46.1.19

604 35. Park SJ, Kang JY, Lee YO. A study on hospital nurses' perception of patient safety culture
605 and safety care activity. J Korean Crit Care Nurs. 2012;5(1):44-55.
606 36. Jeon HW, Kim SA. The effects of fall prevention program on knowledge, burden, and self-
607 efficacy activity related to fall of care facilities workers. J Korean Assoc Local Govern
608 & Adm Stud. 2014;28(3):135-152.
609 37. Zhao YL, Kim H. Older adult inpatient falls in acute care hospitals. Intrinsic, extrinsic and
610 environmental	factors.	J	Gerontol	Nurs.	2015;41(7):29–43.
611 https://doi.org/10.3928/00989134-20150616-05
612 38. Karimi Z, Mousavizadeh A, Rafiei H, Abdi N, Behnammoghadam M, Khastavaneh M, et
613 al. The effect of using olive oil and fish oil prophylactic dressings on heel pressure
614 injury development in critically ill patients. Clinic Cos Invest Derma. 2020;13:59-65.
615 <http://dx.doi.org/10.2147/CCID.S237728>
616 39. Korea Patient Safety reporting & learning system (KOPS). To ensure safe medication
617 administration, device operation is prohibited by anyone other than medical staff
618 [Internet].	KOPS;	c2023	[cited	2024	November	1].
619 https:/[/www.kops.or.kr/portal/psi/patntSafeInfoDetail.do](http://www.kops.or.kr/portal/psi/patntSafeInfoDetail.do)
620 40. Ginsburg L, Castel E, Tregunno D, Norton PG. The H-PEPSS: an instrument to measure
621 health professionals' perceptions of patient safety competence at entry into practice.
622 BMJ Qual Saf. 2012;21(8):676-684. https://doi.org/10.1136/bmjqs-2011-000601
623 41. Halabi O, Scholtes B, Voz B, Gillain N, Durieux N, Odero A, Petre B. “Patient participation”
624 and related concepts: A scoping review on their dimensional composition. Patient Edu
625 Couns. 2019;103(1):5-14. https://doi.org/10.1016/j.pec.2019.08.001
626 42. Oxelmark L, Ulin K, Chaboyer W, Bucknall T, Ringdal M. Registered nurses’ experiences
627 of patient participation in hospital care: Supporting and hindering factors patient
628 participation	in	care.	Scand	J	Car	Sci.	2018;32(2):612-621.
629 https://doi.org/10.1111/scs.12486

630

631

# 632 Figure 1. Flow of the study

633

634

635

636

# Table 1. General Characteristics of the Participants (N=549)

| Characteristics | Categories | Total (n=549) | EFA (n=300) | CFA (n=249) | t / χ^2^ (*p*) |
| --- | --- | --- | --- | --- | --- |
|  |  | N (%) | N (%) | N (%) |  |
| Gender | Man | 183 (33.3) | 99 (33.0) | 84 (33.7) | 0.18  (.856) |
|  | Woman | 366 (66.7) | 201 (67.0) | 165 (66.3) |  |
| Age (year) | 19～29 | 90 (16.4) | 55 (18.3) | 35 (14.1) | 0.13  (.898) |
|  | 30～39 | 204 (37.2) | 101 (33.7) | 103 (41.4) |  |
|  | 40～49 | 150 (27.3) | 82 (27.3) | 68 (27.3) |  |
|  | 50～59 | 47 (8.6) | 32 (10.7) | 15 (6.0) |  |
|  | ≧60 | 58 (10.6) | 30 (10.0) | 28 (11.2) |  |
| Marital status | Married | 292 (53.2) | 149 (49.6) | 143 (57.4) | 1.81  (.070) |
|  | Single | 257 (46.8) | 151 (50.3) | 106 (42.6) |  |
| Education level | Middle school | 17 (3.1) | 8 (2.7) | 9 (3.6) | -0.04  (.970) |
|  | High school | 109 (19.9) | 62 (20.7) | 47 (18.9) |  |
|  | College | 423 (77.1) | 230 (76.6) | 193 (77.5) |  |
| Job | No | 131 (23.9) | 74 (24.7) | 57 (22.9) | -0.49  (.628) |
|  | Yes | 418 (76.1) | 226 (75.3) | 192 (77.1) |  |
| Underlying disease | No | 349 (63.6) | 186 (62.0) | 52 (20.9) | 0.84  (.402) |
|  | Yes | 200 (36.4) | 114 (38.0) | 197 (79.1) |  |
| Procedure/Surgery | No | 114 (20.8) | 62 (20.7) | 52 (20.9) | 0.06  (.950) |
|  | Yes | 435 (79.2) | 238 (79.3) | 197 (79.1) |  |
| Hospitalization (number) | 1 | 190 (34.6) | 99 (33.0) | 91(36.5) | -0.06  (.950) |
|  | 2～3 | 262 (47.7) | 152 (50.7) | 110 (44.2) |  |
|  | 4～5 | 58 (10.6) | 30 (10.0) | 28 (11.2) |  |
|  | 6～9 | 24 (4.4) | 10 (3.3) | 14 (5.6) |  |
|  | ≧10 | 15 (2.7) | 9 (3.0) | 6 (2.4) |  |
| Medical department | Medicine | 316 (57.6) | 178 (59.3) | 138 (55.4) | -1.12  (.263) |
|  | Surgery | 98 (17.9) | 54 (18.0) | 44 (17.7) |  |
|  | Rehabilitation | 21 (3.9) | 10 (3.4) | 11 (4.4) |  |
|  | Orthopedics | 76 (13.8) | 38 (12.7) | 38 (15.3) |  |
|  | Others | 38 (6.9) | 20 (6.7) | 18 (7.2) |  |
| Length of hospital stay (day) | ≤5 | 208 (37.9) | 116 (48.7) | 92 (36.9) | -0.87  (.383) |
|  | 6～15 | 282 (51.4) | 155 (51.7) | 127 (51.0) |  |
|  | ≥16 | 59 (10.7) | 29 (9.7) | 30 (13.0) |  |
| Patient safety incident | No | 469 (85.4) | 257 (85.7) | 212 (85.1) | -0.17  (.862) |
|  | Yes | 80 (14.6) | 43 (14.3) | 37 (14.9) |  |
| Patient safety education | No | 145 (26.4) | 85 (28.3) | 60 (24.1) | -1.12  (.263) |
|  | Yes | 404 (73.6) | 215 (71.7) | 189 (75.9) |  |

EFA=Exploratory factor analysis; CFA=Confirmatory factor analysis; M=Mean; SD=Standard deviation.

637

638

639

# Table 2. Result of Confirmatory Factor Analysis (N=249)

| Factor | Item | β | B | SE | C.R. | AVE | CR |
| --- | --- | --- | --- | --- | --- | --- | --- |
| Factor 1 | xa1 | .44 | 1.00 |  |  | .48 | .82 |
|  | xa2 | .59 | 1.64 | 0.27 | 6.05 |  |  |
|  | xa3 | .62 | 1.60 | 0.26 | 6.19 |  |  |
|  | xa4 | .63 | 1.68 | 0.27 | 6.24 |  |  |
|  | xa6 | .58 | 1.55 | 0.26 | 6.01 |  |  |
| Factor 2 | xb1 | .61 | 1.00 |  |  | .51 | .84 |
|  | xb3 | .63 | 1.00 | 0.12 | 8.36 |  |  |
|  | xb5 | .66 | 1.10 | 0.13 | 8.74 |  |  |
|  | xb6 | .43 | 0.67 | 0.11 | 6.15 |  |  |
|  | xb8 | .60 | 1.01 | 0.13 | 8.12 |  |  |
| Factor 3 | xc1 | .65 | 1.00 |  |  | .57 | .90 |
|  | xc2 | .71 | 1.03 | 0.11 | 9.84 |  |  |
|  | xc3 | .73 | 1.11 | 0.11 | 10.10 |  |  |
|  | xc5 | .71 | 0.96 | 0.10 | 9.76 |  |  |
|  | xc8 | .67 | 0.96 | 0.10 | 9.29 |  |  |
|  | xc9 | .68 | 0.99 | 0.10 | 9.51 |  |  |
|  | xc11 | .64 | 0.87 | 0.10 | 8.92 |  |  |
| Factor 4 | xd2 | .67 | 1.00 |  |  | .52 | .84 |
|  | xd4 | .65 | 1.01 | 0.11 | 9.28 |  |  |
|  | xd7 | .59 | 0.90 | 0.11 | 8.49 |  |  |
|  | xd11 | .67 | 1.05 | 0.11 | 9.54 |  |  |
|  | xd14 | .52 | 0.75 | 0.10 | 7.67 |  |  |
| Factor 5 | xe3 | .65 | 1.10 |  |  | .51 | .80 |
|  | xe6 | .65 | 1.01 | 0.11 | 9.13 |  |  |
|  | xe8 | .63 | 1.10 | 0.12 | 8.90 |  |  |
|  | xe9 | .57 | 0.86 | 0.11 | 8.15 |  |  |
| Factor 6 | xf2 | .62 | 1.00 |  |  | .57 | .84 |
|  | xf5 | .70 | 1.18 | 0.14 | 8.70 |  |  |
|  | xf8 | .60 | 1.00 | 0.13 | 7.76 |  |  |
|  | xf9 | .62 | 1.01 | 0.13 | 7.96 |  |  |
| Factor A ⟷ Factor B | | Φ | SE | Φ-2.00×SE | Φ+2.00×SE | | |
| Factor 1 ⟷ Factor 2 | | 0.94 | 0.02 | 0.89 | 0.98 | | |
| Factor 1 ⟷ Factor 3 | | 0.91 | 0.03 | 0.86 | 0.96 | | |
| Factor 1 ⟷ Factor 4 | | 0.96 | 0.03 | 0.91 | 1.01 | | |
| Factor 1 ⟷ Factor 5 | | 0.99 | 0.03 | 0.94 | 1.04 | | |
| Factor 1 ⟷ Factor 6 | | 0.82 | 0.02 | 0.78 | 0.86 | | |
| Factor 2 ⟷ Factor 3 | | 0.96 | 0.04 | 0.89 | 1.03 | | |
| Factor 2 ⟷ Factor 4 | | 0.98 | 0.03 | 0.91 | 1.04 | | |
| Factor 2 ⟷ Factor 5 | | 0.96 | 0.03 | 0.89 | 1.02 | | |
| Factor 2 ⟷ Factor 6 | | 0.91 | 0.03 | 0.86 | 0.97 | | |
| Factor 3 ⟷ Factor 4 | | 0.95 | 0.04 | 0.87 | 1.02 | | |
| Factor 3 ⟷ Factor5 | | 0.95 | 0.04 | 0.88 | 1.02 | | |
| Factor 3 ⟷ Factor 6 | | 0.84 | 0.03 | 0.77 | 0.90 | | |
| Factor 4 ⟷ Factor 5 | | 0.96 | 0.04 | 0.89 | 1.03 | | |
| Factor 4 ⟷ Factor 6 | | 0.90 | 0.03 | 0.84 | 0.96 | | |
| Factor 5 ⟷ Factor 6 | | 0.85 | 0.03 | 0.80 | 0.91 | | |
| Criteria | | Whether [Φ ± 2.00×SE] includes 1.00 | | | | | |

β=Standardized coefficient; B=Unstandardized coefficient; SE=Standard error; C.R.=Critical ratio; AVE=Average variance extracted; CR=Construct reliability; Φ=Correlation; Factor 1=Patient identification; Factor 2=Medication error prevention; Factor 3=Fall prevention; Factor 4=Infection prevention; Factor 5=Life safety compliance; Factor 6=Information sharing.

640

# Table 3. Model Fit Results (N=249)

Research model	663.48	390	<.001	1.70	.91	.90	.047

Model

χ2

df

*p*	χ^2^/df	CFI	TLI	SRMR	RMSEA

(90% CI)

Alternative model Ⅰ	706.79	405	<.001	1.74	.90	.89	.048

Alternative model Ⅱ	671.71	399	<.001	1.68	.91	.90	.047

.053 (.046∼.060)

.055 (.048∼.061)

| AIC | BIC |
| --- | --- |
| 813.48 | 1077.29 |
| 826.79 | 1037.84 |
| 803.71 | 1035.86 |

.052 (.046∼.059)

CFI=Comparative fit index; TLI=Tuker-lewis index; SRMR=Squared root mean square residual; RMSEA=Root mean square error of approximation; AIC=Akaike Information Criterion; BIC=Bayesian Information Criterion.

641

642

|  |  |  |
| --- | --- | --- |
| Figure 2A. Research model (1-dimensional six factor) | Figure 2B. Alternative model Ⅰ (1-dimensional single factor) | Figure 2C. Alternative model Ⅱ (2-dimensional six factor) |

Factor 1=Patient identification; Factor 2=Medication error prevention; Factor 3=Fall prevention; Factor 4=Infection prevention; Factor 5=Life safety compliance; Factor 6=Information sharing.

# Figure 2. Research model

643	**Appendix 1. Patient safety experience Scale**

| No. | | Items | strongly disagree | disagree | agree | strongly agree |
| --- | --- | --- | --- | --- | --- | --- |
| Patient identification | 1 | I was informed about the patient identification procedures carried out in the hospital. | ① | ② | ③ | ④ |
|  | 2 | I carefully checked whether the medical staff checked my name and registration number (or date of birth) during the medication/examination/surgery process. | ① | ② | ③ | ④ |
|  | 3 | When I received the patient identification bracelet, I put it on after checking that the name was correct. | ① | ② | ③ | ④ |
|  | 4 | I notified the medical staff when my patient identification bracelet was damaged and my name and registration number could not be verified. | ① | ② | ③ | ④ |
|  | 5 | I checked that the medication label attached to the injection matched my name. | ① | ② | ③ | ④ |
| Prevention of medication errors | 6 | I received a thorough explanation from my medical team about the purpose of the medication and how to take it. | ① | ② | ③ | ④ |
|  | 7 | I immediately informed the medical staff when the medication (injection, etc.) was not administered properly. | ① | ② | ③ | ④ |
|  | 8 | When my medications were changed, I was given a full explanation of the reasons for the change. | ① | ② | ③ | ④ |
|  | 9 | If I am not sure exactly how to take the medication (dosage, time, etc.), I ask the medical staff for explanation. | ① | ② | ③ | ④ |
|  | 10 | If I experienced any unusual symptoms (shortness of breath, dizziness, etc.) after taking the medication, I immediately informed the medical staff. | ① | ② | ③ | ④ |
| Fall prevention | 11 | I received regular training from medical staff on fall prevention. | ① | ② | ③ | ④ |
|  | 12 | I was instructed to notify the medical team promptly if I fell (falling out of bed, falling on the floor, etc.). | ① | ② | ③ | ④ |
|  | 13 | I have been given ample instructions on how to raise and lower the bed side rails. | ① | ② | ③ | ④ |
|  | 14 | When I had difficulty moving on my own, I asked the medical staff for help. | ① | ② | ③ | ④ |
|  | 15 | I wear shoes that have a firm, non-slip sole and fit my feet well. | ① | ② | ③ | ④ |
|  | 16 | If I use a wheelchair, I am instructed to sit in the wheelchair seat after securing the wheels. | ① | ② | ③ | ④ |
|  | 17 | I was careful not to trip over the IV line when receiving an IV. | ① | ② | ③ | ④ |
| Infection prevention | 18 | I received a sufficient explanation of infection prevention guidelines (hand washing, wearing a mask, etc.) while hospitalized. | ① | ② | ③ | ④ |

35

644

|  | 19 | I ensured that medical staff performed hand hygiene before administering treatment/medication. | ① | ② | ③ | ④ |
| --- | --- | --- | --- | --- | --- | --- |
|  | 20 | I washed my hands with soap and water after blowing my nose or coughing. | ① | ② | ③ | ④ |
|  | 21 | In the hospital room, I separated general waste and medical waste (swabs, gauze, etc.) and threw them in the trash can. | ① | ② | ③ | ④ |
|  | 22 | Rather than staying in one position while awake, I changed positions periodically. | ① | ② | ③ | ④ |
| Life safety compliance | 23 | When I use medical devices (beds, wheelchairs, crutches, etc.), I use them in the manner instructed. | ① | ② | ③ | ④ |
|  | 24 | I checked the location of the call bell in the hospital room and bathroom. | ① | ② | ③ | ④ |
|  | 25 | I checked the location of the evacuation facilities (guide lights, guide signs, evacuation routes, etc.). | ① | ② | ③ | ④ |
|  | 26 | I immediately reported to the medical staff if any medical  device (drug injector, monitor, etc.) malfunctioned (alarm, etc.) or suddenly turned off. | ① | ② | ③ | ④ |
| Information sharing | 27 | I informed the medical staff if I had any discomfort related to treatment. | ① | ② | ③ | ④ |
|  | 28 | I have received an explanation of the effects and side effects of the examination/treatment/procedure and have  signed a consent form. | ① | ② | ③ | ④ |
|  | 29 | If I had any questions about the examination/treatment/procedure, I asked the medical staff. | ① | ② | ③ | ④ |
|  | 30 | I carefully read and checked the materials provided by the medical staff (hospital life guide, booklets, etc.). | ① | ② | ③ | ④ |

645

36
