# Supplementary material for: Development and internal validation of the patient safety experience scale for inpatients
Source: PLoS One. 2025 Oct 7;20(10):e0332133. doi: 10.1371/journal.pone.0332133 (PMC12503294; doi:10.1371/journal.pone.0332133)
Supplement: Attachment — Submitted filename: Response to reviewer_0729.docx [file pone.0332133.s004.docx]

**Dea****r Editor and Reviewers,**

We wish to thank you for your thoughtful comments and valuable feedback on the manuscript originally titled, “**Development and internal validation of the Patient Safety Experience Scale for inpatients**”.

We have modified the manuscript according to your suggestions, rewriting and rephrasing sections to improve clarity, adding further information, and explaining in detail the points that were previously vague. For your convenience, we have set the revisions in the manuscript in red. We believe that the revised version of this paper will interest the readership of the PLOS ONE.

We have seriously considered the reviewers’ comments and carefully revised the manuscript. Please find our responses to the reviewer’s comments point by point below.

-----------------------------------------------------------------------------------------------------

**Reviewer 1’s comment**

**Point 1:** Please provide the IRB approval number, as it’s ciphered. please include the ethical approval forms as a supporting document.

- **Response:** Thank you for your comments regarding ethical considerations. We have provided the IRB approval number and included it in the text.
- **Line 261-262:** Prior to data collection, approval was obtained from the Institutional Review Board (IRB No: SYU 2023-12-008-003).

**Point 2:** It would be valuable to summarize the limitations identified in the study, if available. Also, adding a section of recommendations would help encourage researchers to conduct further interventional studies to enhance the validity of the instrument. Additionally, it may also be beneficial to investigate other aspects related to the patient safety experience that could be included in the scale.

- **Response:** We appreciate your thoughtful review. In response, we have revised the manuscript by organizing the Limitations and Recommendations sections under clear subheadings.
- **Line 506-520:** **Limitations.** This study has several limitations that should be considered when interpreting the findings. First, the sample consisted primarily of young, female, highly educated surgical patients from five hospitals located in Seoul and its metropolitan area, which may limit the generalizability of the findings to broader inpatient populations, particularly older adults or those with chronic illnesses. As these demographic and clinical traits may influence how patients perceive and report their safety experiences—potentially contributing to higher overall scale scores—caution should be exercised when applying the findings to broader populations. Second, although the average score of the developed scale was relatively high (3.32 out of 4), it did not capture safety experiences in special clinical contexts, such as blood transfusions or care for high-risk patients. Third, one item assessing whether medical staff offered patient safety education may be variably interpreted depending on patients’ individual characteristics and levels of health literacy. Finally, the confirmatory factor analysis revealed high correlations between latent variables, suggesting that discriminant validity was not fully secured, indicating the need for conceptual clarification of the sub-factors.
- **Line 539-548: Implications and recommendations.** To enhance the external validity and broader applicability of the PSES, future studies should include more diverse inpatient populations, such as older adults, individuals with chronic conditions, and those from varied socioeconomic and educational backgrounds. Additionally, intervention-based research is recommended to assess the scale’s sensitivity to changes following safety-enhancing initiatives. Development of condition-specific safety education materials and adaptive questionnaire formats (e.g., branching logic based on patient characteristics) may further improve patient understanding and engagement. Lastly, future research should explore the establishment of clinically meaningful thresholds and normative data by patient group, diagnosis, or care unit, and conduct concept analyses to further clarify and refine the theoretical boundaries of patient safety experience.

**Reviewer 2’s comment**

**Point 1:** This paper reports only the internal validation of the scale, so I think it would be appropriate to mention that in the title.

- **Response:** Thank you for your comments on changing the title to fit the overall structure of the paper. Based on the reviewers' comments, the title has been changed as follows.
- **Revised manuscript (p1):** Development and internal validation of the Patient Safety Experience Scale for inpatients

**Point 2:** The reason to impose an age limit of 65 years was not explained, and this is unexpected because most hopitalized patients are well over 65 year-old. This is a severe limitation of the application of the scale and deverves a justification.

- **Response:** We sincerely appreciate the reviewer's comments and appreciate the in-depth review for the validity of the study. As the reviewer said, patients aged 65 years or older account for a high proportion of hospitalized patients and are a group with high patient safety risks. Nevertheless, the reason for setting the age limit of 65 years in this study is as follows. Cognitive decline frequently occurs in patients aged 65 years or older (Yuan et al., 2021; Chen et al., 2023), and it was judged that this could affect the reliability and validity of self-report measurement tools (Prusaczyk et al., 2017; Nichols et al., 2023). In addition, in the early stage of measurement tool development, it is important to secure internal validity by targeting a group with relatively uniform response ability, so participants of that age group were selected to select a relatively uniform group. Therefore, this study is significant in establishing the basic validity of a patient safety experience measurement tool for adults aged 19-65, and is expected to serve as a foundation for the development of a comprehensive measurement tool including elderly patients in the future. The literature of the study presented in the response is presented below.
- **Line 192-197:** The inclusion criteria were as follows: In order to secure internal validity by targeting a group with relatively uniform response ability, patients aged 19 to 65 years, patients hospitalized for at least 3 days, able to understand the purpose of the study, and who provided informed consent. Exclusion criteria included patients hospitalized on the same day, patients visiting the outpatient clinic, and those with cognitive problems or inability to communicate.
- Yuan, L., Zhang, X., Guo, N., Li, Z., Lv, D., Wang, H., ... & Wu, X. (2021). Prevalence of cognitive impairment in Chinese older inpatients and its relationship with 1-year adverse health outcomes: a multi-center cohort study. BMC geriatrics, 21(1), 595.
- Chen, P., Cai, H., Bai, W., Su, Z., Tang, Y. L., Ungvari, G. S., ... & Xiang, Y. T. (2023). Global prevalence of mild cognitive impairment among older adults living in nursing homes: a meta-analysis and systematic review of epidemiological surveys. Translational Psychiatry, 13(1), 88.
- Prusaczyk, B., Cherney, S. M., Carpenter, C. R., & DuBois, J. M. (2017). Informed consent to research with cognitively impaired adults: transdisciplinary challenges and opportunities. Clinical Gerontologist, 40(1), 63-73.
- Nichols, E., Ng, D. K., Hayat, S., Langa, K. M., Lee, J., Steptoe, A., ... & Gross, A. L. (2023). Measurement differences in the assessment of functional limitations for cognitive impairment classification across geographic locations. Alzheimer's & Dementia, 19(5), 2218-2225.

**Point 3:** Line 123: details should be given on how were the 90 items of the initial scale created.

- **Response:** Thank you for your comments on the initial draft of the questions. We have reviewed and rewritten the drafted parts of the questions.
- **Line 126-133:** Initial items were developed based on the conceptual attributes of the patient safety experience through literature review and in-depth interviews. According to Devellis and Thorpe [19], the more initial items, the better, and they should be more than 50% more than the number of questions in the final scale. Referring to this recommendation, this study composed the number of initial items by component from 12 to 18. A total of 90 items were generated, consisting of 12 items for patient identification, 14 for the prevention of medication errors, 16 for fall prevention, 16 for infection prevention, 14 for compliance with safety in daily life, and 18 for information sharing.

**Point 4:** Line 218: I believe the comma between the words "value" and "and" should be deleted.

- **Response:** Thank you for your careful review of the sentence structure. We have rewritten it by removing the comma between the words "value" and "and".
- **Line 227-228:** CFA was conducted to evaluate the model fit. The model was considered acceptable when the ratio of the chi-square (χ²) value to degrees of freedom was 3 or less.

**Point 5:**  Line 224: the terminology adopted in factor analysis is to name coefficients by loadings, so I think the term factor loadings should be used throughout the text.

- **Response:** We sincerely appreciate your help in organizing the words that should be written and used in factor analysis. Based on the review comments, we have changed the word that We wanted to name coefficients as loadings to “factor loadings” in general.
- **Line 232-238:** Standardized factor loadings (β) and modification indices (MI) were reviewed to revise the model, if necessary. To verify the convergent validity, the following criteria were applied: standardized factor loading (β) ≥ 0.50, average variance extracted (AVE) ≥ 0.50, and construct reliability (CR) ≥ 0.70 [27]. Discriminant validity was assessed using the confidence interval of the correlation coefficient (Φ ± 2.00 × SE); if the interval did not include 1.00, the constructs were considered distinct, indicating that discriminant validity was established [26].

**Point 6:** Line 204-208: It appears to me that this paragraph would be better placed at the beginning of the Results section.

- **Response:** Thank you for your feedback on the smooth and concise refinement of sentences and the harmonious connection between paragraphs. Based on your feedback, We have modified it to place the relevant content at the beginning of the research results section so that the connection between sentences flows naturally.
- **Line 276-278:** The survey took approximately 15–20 min to complete. In total, 551 participants responded: 149 via paper-based survey and 402 via online survey. After excluding two incomplete responses, data from 549 participants were included in the final analysis.

**Point 7:** Line 215: could the authors explain why the promax rotation was used, instead of an orthogonal rotation. I found this difficult to understand because there are no compelling arguments against the independence of the dimensions.

- **Response:** We would like to thank you for your feedback on the Promax rotation method for exploratory factor analysis of data analysis methods. In this study, the Promax method among oblique rotations was selected considering the possibility of correlation between factors during exploratory factor analysis. Since the scale related to patient safety experience includes various sub concepts that are closely connected to each other in actual clinical and organizational contexts, it was judged that there is a high possibility of correlation between them. Orthogonal rotation assumes complete independence (correlation=0) between factors, but various previous studies have reported that factors related to patient experience actually influence each other. In papers on the development of tools dealing with psychosocial and clinical complex concepts, oblique rotation (Promax, Oblimin, etc.) that allows correlation between factors is suggested to be more valid. In fact, scales with multidimensional properties such as patient experience, patient safety, quality of life, and health-related attitudes are often verified on the premise of correlation between factors. Accordingly, it was difficult to accept the assumption that individual factors were 'completely independent', and it was interpreted that various aspects of each factor were realistically connected. Therefore, in this study, we selected rectangular rotation (Promax) that can reflect the relationship between factors based on theoretical, empirical, and statistical grounds, and judged that this is more appropriate for measuring the complex construct called patient safety experience.
- **Line 221-226:** EFA was performed using the maximum likelihood method, as the assumption of normality was satisfied in the item analyses, making it a statistically robust approach. To allow for correlations among latent factors, Promax rotation—an oblique rotation technique widely used in large datasets—was applied, which generates results based on initial orthogonal rotation outcomes [21]. Items with factor loadings below .50 or communalities below .30 were considered for deletion [25].

**Point 8:** Line 219-223: please review the writing of this paragraph. It presents the criteria for the definition of adequate fit indices, but as it stands might be confused by the results of the analysis of the fit indices.

- **Response:** We sincerely appreciate your feedback on sentences that may confuse readers about the fit indices. We have deleted sentences that may cause confusion by listing multiple indices and criteria in one paragraph, making it difficult for readers to distinguish between results and criteria, or by mixing criteria and actual analysis results, and have revised each index into shorter sentences to clearly distinguish between criteria (cut-off values) and results (observed values).
- **Line 227-238:** CFA was conducted to evaluate the model fit. The model was considered acceptable when the ratio of the chi-square (χ²) value to degrees of freedom was 3 or less. Goodness-of-fit thresholds were defined as follows: SRMR ≤ 0.08, RMSEA ≤ 0.08 (with a 90% confidence interval upper bound ≤ 0.10), CFI ≥ 0.90, and TLI ≥ 0.90. The final model showed acceptable fit indices: SRMR = 0.06, RMSEA = 0.07 (90% CI: 0.05–0.09), CFI = 0.92, and TLI = 0.91 [23]. Standardized factor loadings (β) and modification indices (MI) were reviewed to revise the model, if necessary. To verify the convergent validity, the following criteria were applied: standardized factor loading (β) ≥ 0.50, average variance extracted (AVE) ≥ 0.50, and construct reliability (CR) ≥ 0.70 [27].

**Point 9:** Line 228: please explain the correlation coefficient for discriminant validity.

- **Response:** Thank you for your feedback that we need to provide an explanation of discriminant validity so that readers can understand it clearly. We have added more information about discriminant validity.
- **Line 235-238:** Discriminant validity was assessed using the confidence interval of the correlation coefficient (Φ ± 2.00 × SE); if the interval did not include 1.00, the constructs were considered distinct, indicating that discriminant validity was established [26].

**Point 10:** Line 309: I believe that instead of "were 1.00" should be 'include 1.00".

- **Response: We sincerely** thank you for your precise comments and reviews on the discriminant validity. We have revised the relevant sentence. We would like to express my sincere gratitude once again for your review that allows me to see the numerical analysis in more detail.
- **Line 323-325:** However, some confidence intervals for the inter-factor correlation coefficients include 1.00, suggesting that discriminant validity was only partially supported (Table 2).

**Point 11:** Line 318: please explain the two-dimensional 6-factor structure proposed. There was no mention to the two-distinct, separate, undelying latent factors, and figure 2C shows only a single factor. In my opinion, the difference between the research model and model II is that in the former the factors are allowed to be correlated, while in the latter they are assumed to be independent.

- **Response:** Thank you for your feedback on the model of this study. We acknowledge the confusion caused by the terminology “two-dimensional” in the description of Alternative Model II. Upon your suggestion, we have revised the terminology and clarified the structural assumptions of the model accordingly. To clarify, Alternative Model II does not introduce two distinct, higher-order latent factors. Rather, it maintains the same six first-order latent factors as the initial research model. The key distinction lies in the correlation structure: in the initial model (Fig 2A), correlations among the six factors are freely estimated, whereas in Alternative Model II (Fig 2C), the six factors are specified as mutually independent, with all inter-factor covariances fixed to zero. In this context, the term “two-dimensional” was inappropriately used and has now been corrected throughout the manuscript to avoid misinterpretation. We have replaced it with “uncorrelated six-factor,” and revised the corresponding explanation in both the text and figure legends. We appreciate your attention to this detail, which helped us improve the clarity and accuracy of our model description.
- **Line 330-340:** The initial research model (Fig 2A) assumed a structure comprising six first-order latent factors that were allowed to correlate freely. However, discriminant validity was not fully supported due to high inter-factor correlation coefficients. When such correlations compromise discriminant validity, it is recommended to either simplify the model to a single-factor structure or specify the factors as mutually independent [27]. Therefore, two alternative models were tested using CFA: Alternative Model I, which specified a unidimensional single-factor structure (Fig 2B); and Alternative Model II, which retained the six-factor structure but constrained all inter-factor correlations to zero (Fig 2C). Unlike the original model that allows correlations among latent factors, Alternative Model II assumes that the six factors are orthogonal, thereby representing them as conceptually distinct and statistically independent constructs.

**Point 12:** Still on the same subject, if I am right and model II represents a data structure where the six factors are assumed to be independent, how this aligns with the use of an oblique rotations?

- **Response:** We sincerely appreciate the in-depth opinions and feedback on the data analysis methods of the reviewers. We tried to write the research method we actually performed faithfully. This study assumed that there was a possibility of correlation between factors in the initial model, so we analyzed it using promax rotation in EFA. After confirming EFA, we checked the correlation coefficients between the six latent variables through CFA with another sample, and most of them showed high correlations of .90 or higher. Based on these results, if the correlation between latent variables is high and discriminant validity is not secured, an alternative model can be considered, so we additionally verified the independent factor structure (Model II) in the confirmation stage based on the analysis results. We described the main text according to the procedure of this study, and modified it clearly by deleting confusing terms (two-dimensional). We sincerely appreciate your valuable opinions for improving the quality of the paper.

**Point 13:** Line 388: I believe there is some text missing in the middle of the sentence.

- **Response:** We have reviewed the relevant sentence based on the reviewer's review, and have confirmed that the content of the sentence is missing. We have revised and rewritten the relevant sentence. Thank you for carefully checking the errors in the sentence and the text.
- **Line 414-419:** Factor 2 was “prevention of medication errors,” which comprises five items, such as receiving explanations from medical staffs about the purpose and effects of medications, and informing when the injection fluids are not administered. This finding is consistent with that of previous studies involving hospitalized patients, which emphasized patient safety behaviors, such as being informed about drug efficacy, administration methods, side effects, and precautions, as well as notifying healthcare providers in the event of abnormal infusion rates [16,31].

**Point 14:** Table 1: unless the patient sex was self-reported, instead of Gender the appropriate term would be Sex.

- **Response:** Thank you for your feedback. Based on your feedback, we have changed the content of gender in the general characteristics of the table.
- **Revised manuscript (p15~16): Table 1.** Gender 🡪 Sex

**Point 15:** Appendix 1. How should a patient score item 16 if the patient never used a wheelchair? From my point of view, the weak part of the manuscript is the Discussion section, as the results raise a number of questions that were not approached by the authors. Here are my suggestions to improve that section:

- **Response:** We sincerely appreciate your insightful comments. We appreciate your careful review and valuable suggestions regarding the clarity of item 16 and the limitations of the Discussion section. Regarding item 16, we recognize that it may be ambiguous for patients who have never used a wheelchair. Nevertheless, it was adopted after a validity assessment by experts, as awareness of safe wheelchair use can also be considered as ‘experience’. In the future, we will conduct a study to revise the questionnaire to include guidance on items that may not apply to all respondents, treat ‘not applicable (N/A)’ as missing values during the scoring process, and consider using appropriate statistical techniques such as mean imputation or full information maximum likelihood based on the results of the analysis.

**Point 16:** The sample characteristics should be justified and whether the results may have been impacted by that specific sample should be discussed. This is because the sample characteristics are not what would be expected from an inpatient population. They are mostly young adults, females, most with university educations, surgical patients, the majority with previous hospitalizations. Therefore, the sample characteristics are not those of a population with chronic illnesses that would be expected from a general hospital-based study.

- **Response:** Thank you very much for your thoughtful comment regarding the representativeness of the sample. We discuss how factors such as age, prior hospitalization experience, and educational background could influence patients’ perceptions of safety, potentially leading to higher mean scores on the scale. We have added a recommendation that future validation studies be conducted in more diverse inpatient populations, including those with chronic conditions, older adults, and patients from different socioeconomic backgrounds, to ensure the broader applicability of the scale. We sincerely appreciate your comment, which led to important improvements in both the clarity and external validity considerations of our study.
- **Line 190-197:** Participants in the main survey were conveniently sampled from five small- and medium-sized hospitals (each with fewer than 300 beds) located in Seoul city and the surrounding metropolitan area. The inclusion criteria were as follows: In order to secure internal validity by targeting a group with relatively uniform response ability, patients aged 19 to 65 years, patients hospitalized for at least 3 days, able to understand the purpose of the study, and who provided informed consent. Exclusion criteria included patients hospitalized on the same day, patients visiting the outpatient clinic, and those with cognitive problems or inability to communicate.
- **Line 506-513:** This study has several limitations that should be considered when interpreting the findings. First, the sample consisted primarily of young, female, highly educated surgical patients from five hospitals located in Seoul and its metropolitan area, which may limit the generalizability of the findings to broader inpatient populations, particularly older adults or those with chronic illnesses. As these demographic and clinical traits may influence how patients perceive and report their safety experiences—potentially contributing to higher overall scale scores—caution should be exercised when applying the findings to broader populations.

**Point 17:** A section discussing the limitations and strenghts of the study needs to be included. Some of the the issues were presented in the Conclusions but a better place would be in the limitations.

- **Response:** Thank you for your valuable comment. We fully agree that limitations and strengths should be clearly addressed in a dedicated section rather than being partially mentioned in the Conclusions. In response, we have revised the manuscript to include a separate and structured discussion of both the limitations and strengths of the study in the 'Limitations' section and 'Implications and Recommendations' section.

**Point 18:** Also, the authors should discuss whether in their opinion this scale is ready to be applied in clinical practice and, in the afirmative, when, to whom and by whom the scale should be applied. In addition, some leads should be given on how to interpret the scale scores, that is, what is a clinical significant score, should subscale scores be interpreted and how.

- **Response:** Thank you for your feedback on the study results of the sample. We have described the results along with a brief description of the sample.
- **Line 532-538:** The PSES was designed for ease of administration and interpretation, making it suitable for integration into routine inpatient care. It can be administered by patient safety officers, clinical quality teams, or researchers to assess patients’ perceived safety experiences during or immediately after hospitalization. Although clinical cutoff scores have not yet been established, the total score (average of 30 items on a 4-point Likert scale) serves as an overall indicator of patient-perceived safety, while subscale scores can help identify specific areas for targeted quality improvement.

**Point 19:** Following the limitations section, suggestions for future research should be proposed. Again, some were presented in the Conclusions section, but a better place would be in the Discussion**.**

- **Response:** Thank you for your careful review comments that took into account the context of the study. Based on your comments, we have written a section on limitations and added additional recommendations that fit the content.
- **Line 539-548:** To enhance the external validity and broader applicability of the PSES, future studies should include more diverse inpatient populations, such as older adults, individuals with chronic conditions, and those from varied socioeconomic and educational backgrounds. Additionally, intervention-based research is recommended to assess the scale’s sensitivity to changes following safety-enhancing initiatives. Development of condition-specific safety education materials and adaptive questionnaire formats (e.g., branching logic based on patient characteristics) may further improve patient understanding and engagement. Lastly, future research should explore the establishment of clinically meaningful thresholds and normative data by patient group, diagnosis, or care unit, and conduct concept analyses to further clarify and refine the theoretical boundaries of patient safety experience.

**Point 20:** Finally, the conclusions should be limited to the interpretantion of the more relevant aspects of the research. I strongly recommend that the authors clearly emphsize that only the results of the internal validation were presented, and further studies evaluating criterion validity, responsiveness, generalizability and transcultural validity are necessary.

- **Response:** Thank you for this important suggestion. We agree that the conclusions should be limited to the scope of the present study and clearly reflect the boundaries of the validation performed. In response, we have revised the Conclusions section to emphasize that this study presents only the results of internal validation, including internal consistency and construct validity.
- **Line 551-559:** This study developed the Patient Safety Experience Scale (PSES), a 30-item, uncorrelated six-factors, to quantitatively assess inpatients’ safety-related experiences. Grounded in internationally recognized patient safety frameworks, the scale demonstrated acceptable internal consistency and construct validity through a rigorous development and validation process. Designed for ease of use in clinical settings, the PSES provides a structured and patient-centered approach to capturing safety perceptions during hospitalization. However, as this study focused solely on internal validation, further research is required to establish criterion validity, responsiveness, generalizability, and cross-cultural applicability in diverse healthcare environments.

**Editor’s comment**

**Point 1:** Please provide the IRB approval number, as it’s ciphered here.

- **Response:** Thank you for your review comments for a systematic approach to data collection methods. After reviewing the collection procedure of the paper, we tried to describe the collection procedure according to the flow chart to support it, and revised the text with the contents of the paper collection procedure to see the overall research trend.
- **Line 261-262:** Prior to data collection, approval was obtained from the Institutional Review Board (IRB No: SYU 2023-12-008-003).

**Point 2:** It would be valuable to summarize the limitations identified in the study, if available. Also, adding a section of recommendations would help encourage researchers to conduct further interventional studies to enhance the validity of the instrument. Additionally, it may also be beneficial to investigate other aspects related to the patient safety experience that could be included in the scale.

- **Response:** Thank you for your valuable comment. We fully agree that limitations and strengths should be clearly addressed in a dedicated section rather than being partially mentioned in the Conclusions. In response, we have revised the manuscript to include a separate and structured discussion of both the limitations and strengths of the study in the 'Limitations' section and 'Implications and Recommendations' section.
